# CD147 modulates autophagy through the PI3K/Akt/mTOR pathway in human prostate cancer PC-3 cells

**DOI:** 10.3892/ol.2015.2849

**Published:** 2015-01-05

**Authors:** FANG FANG, LIUHANG WANG, SHUFANG ZHANG, QING FANG, FENG HAO, YANMEI SUN, LIANGZHONG ZHAO, SHUANG CHEN, HUIJUAN LIAO, LIGUO WANG

**Affiliations:** 1 Department of Immunology, Jilin Medical College, Jilin, Jilin 132013, P.R. China; 2 Department of Biochemistry, Changchun Medical College, Changchun, Jilin 130021, P.R. China; 3 Department of Biochemistry, Jilin Medical College, Jilin, Jilin 132013, P.R. China; 4 Department of Urology, Affiliated Hospital of Jilin Medical College, Jilin, Jilin 132011, P.R. China

**Keywords:** cluster of differentiation 147, autophagy, prostate cancer, PI3K/Akt/mTOR pathway

## Abstract

The multifunctional glycoprotein cluster of differentiation (CD)147 is highly expressed on the cell surface of the majority of cancer cells, and promotes tumor invasion, metastasis and growth. However, the role of CD147 in autophagy has not yet been explored in prostrate cancer cells. In the present study, prostate cancer PC-3 cells were cultured under starvation conditions, and the expression level of CD147 gradually increased. Therefore, RNA interference was used to inhibit CD147 expression, in order to investigate the biological role of this glycoprotein in autophagy progression. Autophagic activity was monitored by the changes in green fluorescent protein-light chain 3 (GFP-LC3) location and the expression of the autophagy-associated protein LC3-II. It was found that downregulation of CD147 significantly promoted GFP-LC3 puncta formation and the expression of LC3-II. Furthermore, the levels of phosphorylated serine/threonine protein kinase B (p-Akt) and phosphorylated mammalian target of rapamycin (p-mTOR) were significantly decreased, and the level of LC3-II was inversely associated with levels of p-Akt and p-mTOR in cells with downregulated expression of CD147. The results of a trypan blue exclusion assay revealed that starvation-induced cell death was increased in PC-3/shCD147 cells compared with control PC-3/Scramble cells (37.7±6.4 vs. 21.7±5.5%). Together, these results indicate that CD147 may be important in the inhibition of autophagy via the PI3K/Akt/mTOR pathway, which prevents cell death from unrestrained autophagy.

## Introduction

Autophagy is an intracellular lysosomal degradation process that facilitates the proteolytic degradation of cell contents to generate small reusable biomolecules. Autophagy plays a role in a wide variety of physiological and pathological processes, including adaption to starvation (1), embryonic development (2), cell survival and death (3), and tumor suppression (4). Autophagy is more adapted to promoting cell survival in starvation conditions, but unrestrained autophagy can induce progressive consumption of cellular constituents and ultimately lead to cell death (5). The pathways involved in the progression of autophagy are complicated and include the phosphoinositide 3-kinase (PI3K)/serine/threonine protein kinase B (Akt) pathway, which allows cells to inhibit autophagic progression. Activated Akt is a downstream effector of PI3K, which can stimulate the mammalian target of rapamycin (mTOR) to negatively regulate autophagy (6).

Prostate cancer is one of the leading worldwide causes of cancer-associated mortality in human males. Cluster of differentiation (CD)147, also termed extracellular matrix metalloproteinase inducer, is highly expressed on the cell surface of the majority of cancer cells, including prostate cancer cells (7). CD147 has been indicated as a prognostic marker in prostate cancer. Several *in vitro* studies have indicated that CD147 is a multifunctional glycoprotein that inhibits tumor cell anoikis (8), enhances tumor angiogenesis (9), promotes invasion and metastasis (10) and also promotes glycolytic energy metabolism (11). Previous studies revealed that CD147 plays an important role in the invasion and metastasis of prostate cancer by inducing matrix metalloproteinase 2 (MMP2) and MMP9 secretion (12–14). Metastasis and invasion is also regulated by the PI3K/Akt signaling pathway (15). Therefore, it was hypothesized that the PI3K/Akt pathway may be involved in the regulation of autophagy by CD147. The present study investigates the association between CD147 and autophagy, and the potential molecular mechanisms in prostate cancer PC-3 cells.

## Materials and methods

### Cell culture

The human prostate PC-3 cell line, which was provided by the Institute of Biochemistry and Cell Biology, Chinese Academy of Science (Shanghai, China), was maintained in Dulbecco’s modified Eagle’s medium-F12 (Gibco Life Technologies, Carlsbad, CA, USA), supplemented with 10% fetal calf serum, at 37°C and under a mixture of 95% air and 5% CO_2_. To investigate amino acid starvation-induced autophagy, the cells were cultured for 12 h in Earle’s balanced salt solution (EBSS) medium at 37°C, in a 95% air and 5% CO_2_ atmosphere, to induce autophagy as previously described (16). The study was approved the ethics committee of Jilin Medical College (Jilin, China).

### Gene transfection and stable cell line selection

The pSilencer-shCD147 plasmid, which produces CD147 hairpin small interfering RNA, was provided by Dr Liguo Wang (Affiliated Hospital of Jilin Medical College, Jilin, China). The PC-3 cells were seeded in six-well culture plates at a concentration of 5×10^5^ cells/ml for 24 h. Transient transfections were performed in a six-well plate containing serum-free medium, using Lipofectamine™ 2000 reagent (Gibco Life Technologies) and 2 μg of plasmid DNA, according to the manufacturer’s instructions. The G418 antibiotic (1,000 μg/ml) was used to screen for positive clones. The cells that demonstrated low expression of CD147 were termed PC-3/shCD147. PC-3/Scramble negative control cells were prepared by transfecting the pSilencer-scramble plasmid into PC-3 cells as previously described (12).

### Reverse transcription-polymerase chain reaction (RT-PCR) analysis

Total RNA was extracted from cells using TRIzol reagent (Invitrogen, Carlsbad, CA, USA). A one-step RT-PCR was performed for the CD147 gene using a kit from Qiagen GmbH (Hilden, Germany). β-actin was amplified as an internal control. The PCR primers used were: CD147 forward, 5′-AAGGTGGACTCCGACGACCAGTGG-3′ and reverse, 5′-CTTCCGGCGCTTCTCGTAGATGAAG-3′; and β-actin forward, 5′-ATCATGTTTGAGACCTTCAACA-3′ and reverse, 5′-CATCTCTTGCTCGAAGTCCA-3′. The amplified products were separated on a 1% agarose gel for 30 min, followed by ethidium bromide staining.

### Western blot analysis

The cells were washed twice with phosphate-buffered saline (PBS). Subsequently, the cells were lysed with RIPA lysis buffer (Beyotime Institute of Biotechnology, Haimen, Jiangsu, China). The protein concentrations were determined using a bicinchoninic acid kit (Pierce Biotechnology, Inc., Rockford, IL, USA). Equal amounts of the total protein were separated by 12% sodium dodecylsulfate-polyacrylamide gel electrophoresis (SDS-PAGE) and transferred to polyvinylidene difluoride (PVDF) membranes (Millipore, Billerica, MA, US). The membranes were subsequently immunoblotted with the appropriate primary antibody diluted in Tris-buffered saline, containing 0.05% Tween-20 and 5% skimmed dry milk, at 4°C overnight. The following primary antibodies were used: anti-CD147 (cat. no. 3212) and anti-β-actin (cat. no. 1854) rabbit IgG monoclonal antibodies (Epitomics, Burlingame, CA, USA); and anti-light chain 3 (LC3; cat. no. 3868), p-Akt (cat. no. 4060) and p-mTOR (cat. no. 2971) rabbit IgG monoclonal antibodies (Cell Signaling Technology, Inc, Danvers, MA, USA). The membranes were washed and incubated using appropriate secondary horseradish peroxidase-conjugated goat anti-rabbit (1:5,000) antibody (Beyotime Institute of Biotechnology). β-actin was used to confirm equal protein loading. The band density was evaluated by Bio-Rad Quantity One software (Bio-Rad Laboratories, Hercules, CA, USA).

### Green fluorescent protein (GFP)-LC3 transfection

For the quantitative analysis of autophagy, GFP-LC3 puncta formation was quantified as previously described (17). The cells were cultured in six-well plates and transfected with the GFP-LC3 plasmid, using Lipofectamine 2000, following the manufacturer’s protocol. At 24 h post-transfection, the cells were cultured with EBSS medium for 12 h, and the cells were subsequently examined under Nikon fluorescence microscopy (magnification, ×100; 90i; Nikon Corporation, Tokyo, Japan). For each condition, three slides were used and 30 cells were counted per slide.

### Trypan blue exclusion assay

Starvation-induced cell death was evaluated by a trypan blue exclusion assay. In brief, subsequent to PC-3/shCD147 or PC-3/Scramble being cultured in EBSS medium for 12 h, adherent and non-adherent cells were harvested, and resuspended in 100 μl PBS. Subsequent to mixing with 100 μl of 0.8% trypan blue, the cells were counted using a hemocytometer (Beyotime Institute of Biotechnology). The number of dead cells with disrupted membranes, stained blue, out of a total 200 cells was counted in three replicates. Cell death was calculated by the mean percentage of blue cells/total cells.

### Statistical analysis

The data were calculated as the mean ± standard deviation of the mean. One-way analysis of variance was used to compare the significant difference in means between all treatment groups, and a two-sided Student’s t-test was used to compare the means of individual treatments when the primary outcome was statistically significant. P<0.05 was considered to indicate a statistically significant difference. All statistical analyses were performed using SPSS 13.0 software (SPSS, Inc., Chicago, IL, USA).

## Results

### Starvation-induced autophagy was associated with CD147 upregulation

**Figure 1:**
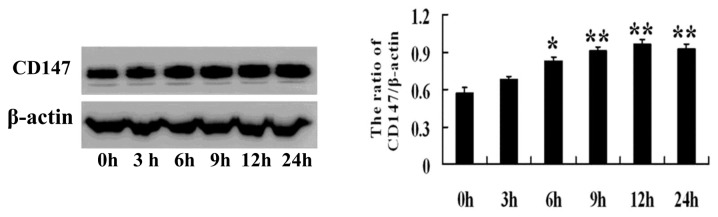
Change in CD147 expression in starvation-induced autophagy in PC-3 cells. CD147 expression was assayed by western blotting. ^*^P<0.05 vs. 0 h, ^**^P<0.01 vs. 0 h. CD147, cluster of differentiation 147.

To evaluate the expression of CD147 under starvation conditions, autophagy was induced in the human prostate cancer PC-3 cell line by culturing cells in amino acid-free EBSS buffer for 0, 3, 6, 9, 12 and 24 h. CD147 protein expression was determined by western blot analysis. The results revealed that CD147 expression gradually increased at 3, 6, 9, 12 and 24 h (Fig. 1). These data demonstrated that CD147 was involved in starvation-induced autophagy in PC-3 cells.

### CD147 inhibits autophagy in PC-3 cells

**Figure 2:**
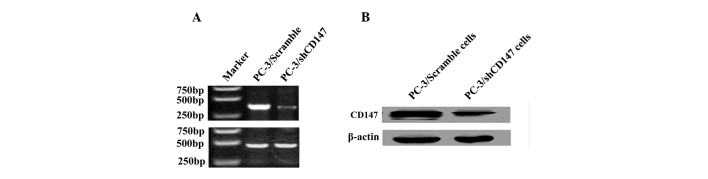
CD147 expression was detected in transfected cells. (A) Reverse transcription-polymerase chain reaction analysis of the CD147 gene level. (B) Western blot analysis of the CD147 protein level. CD147, cluster of differentiation 147; shCD147, short hairpin CD147 RNA.

PC-3 cells stably expressing CD147 or control hairpin RNA were generated by transfecting the cells with the pSilencer-shCD147 plasmid or control pSilencer-scramble plasmid, respectively. Clones positive for the plasmid were screened for using the G418 antibody. The expression of CD147 was evaluated by RT-PCR (Fig. 2A) and western blot analysis (Fig. 2B). The results confirmed that the expression of CD147 was significantly downregulated in the PC-3/shCD147 cells at the mRNA and protein levels.

**Figure 3:**
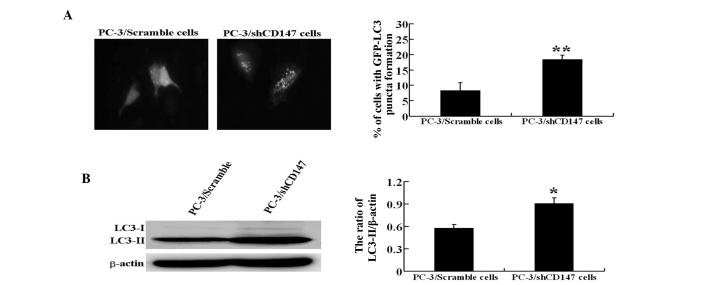
CD147-mediated inhibition of starvation-induced autophagy in PC-3 cells. (A) The autophagosomes in the cells were visualized by observing LC3 puncta under fluroesence microscopy. (B) Quantitated levels of LC3-II from western blot analysis. ^*^P<0.05, ^**^P<0.01 vs. control cells. CD147, cluster of differentiation 147; shCD147, short hairpin CD147 RNA; GFP, green fluorescence protein; LC3, light chain 3.

To confirm the modulation of autophagy by CD147, the autophagosome formation was evaluated using two assays. LC3 is essential for final autophagosome formation and thus serves as an autophagosome marker. First, each experimental stable cell line was transfected with a GFP-LC3 plasmid and the cells were cultured in EBSS medium for 12 h. The formation of autophagosomes was detected by the presence of GFP-LC3 punctate dots. The results revealed that PC-3/shCD147 cells exhibited a higher number of punctate structures compared with PC-3/Scramble control cells (P<0.01) (Fig. 3A).

The endogenous levels of LC3-II most likely indicated the level of autophagy. Therefore, western blot analysis was performed to detect LC3-II expression in PC-3/Scramble and PC-3/shCD147 cells. Notably, during the induction of starvation, increased LC3-II was observed in PC-3/shCD147 cells compared with the control PC-3/Scramble cells (P<0.05), further indicating that autophagy was induced to higher levels in the absence of CD147 (Fig. 3B).

### CD147 inhibits autophagy through the activation of the PI3K/Akt/mTOR pathway

**Figure 4:**
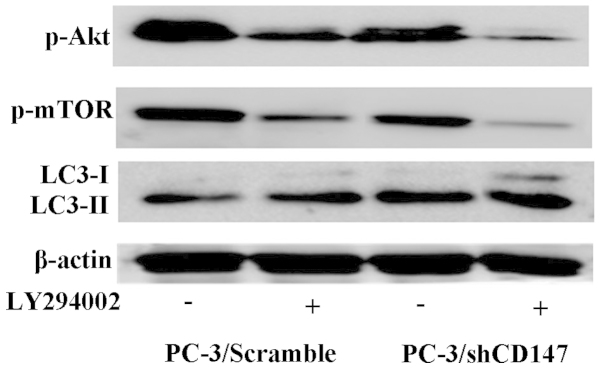
Analysis of the signaling pathway involved in the downregulation of atophagy by CD147. The cells were cultured for 12 h in EBSS medium with or without 20 μM LY294002. Western blot analysis was performed to observe the expression level of p-Akt, p-mTOR and LC3-II. p-Akt, phosphorylated protein kinase B; p-mTOR, phosphorylated mammalian target of rapamycin; LC3, light chain 3; CD147, cluster of differentiation 147; shCD147, short hairpin CD147 RNA.

To investigate the effect of the PI3K/Akt/mTOR pathway in the regulation of autophagy induced by CD147, the expression levels of p-Akt, p-mTOR and LC3-II were examined in the transfected PC-3 cells that were cultured with or without 20 μM LY294002, a specific inhibitor of Class I PI3K. It was found that the expression level of p-Akt and p-mTOR notably decreased in PC-3/shCD147 cells compared with PC-3/Scramble cells cultured without LY294002. In addition, compared with control shRNA-transfected PC-3 cells, PC-3 cells transfected with CD147 shRNA exhibited a significantly decreased level of p-Akt and p-mTOR subsequent to culturing with LY294002. To further assess the effect of the PI3K/Akt/mTOR pathway on autophagy, the levels of LC3-II in transfected PC-3 cells were examined subsequent to culturing with or without LY294002, using western blot analysis. The present results revealed that the inhibition of PI3K activity by LY294002 further promoted the autophagic levels in PC-3/shCD147 cells compared with PC-3/Scramble cells (Fig. 4). These results indicate that the PI3K/Akt/mTOR pathway is involved in the autophagy inhibition caused by CD147.

### CD147 enhances survival of PC-3 cells by inhibiting starvation-induced autophagy

Trypan blue exclusion was used to investigate the starvation-induced cell death in transfected PC-3 cells cultured in EBSS medium for 12 h. The present data indicated that starvation-induced cell death was increased in PC-3/shCD147 cells compared with control PC-3/Scramble cells (37.7±6.4 vs. 21.7±5.5%; P<0.05). These findings indicate that CD147 may enhance the survival of PC-3 cells by inhibiting starvation-induced autophagy.

## Discussion

Autophagy is an intracellular degradation process that is an important component in the regulation of protein homeostasis and is essential for cell survival when cells undergo metabolic stress. Studies investigating the role of autophagy in determining mammalian cell fate remain controversial. Cancer cells tend to reprogram their metabolism machinery to evade cell death. In this context, when the tumor is deficient nutrients, autophagy may aid cancer cells to adapt to changing conditions, preventing their death. However, autophagy in cancer can be a double-edged sword, as excessive autophagy results in cell death (18). This may explain numerous controversial topics associated with the toxicity of autophagy. However, the effects of autophagy appear to be strictly regulated in the cells maintained under starvation conditions.

CD147 is important in tumor biology, inhibiting cancer cell anoikis and promoting invasion and metastasis. Previous studies have indicated that CD147 exerts a broader effect in tumor growth. In this context, the effects of CD147 on starvation-induced autophagy were investigated in the present study, using human prostate cancer PC-3 cells cultured in amino acid-free EBSS buffer. The results revealed that CD147 expression was gradually increased when PC-3 cells were starved for 3, 6, 9 and 12 h. This observation led to the hypothesis that CD147 may regulate autophagy.

LC3 is essential for final autophagosome formation and exists in two forms, a cytosolic form (LC3-I) and a lipid phosphatidylethanolamine-conjugated form (LC3-II), which is inserted into the inner and outer membranes of the growing autophagosome. LC3-II then translocates to the autophagosome membrane, and this process is essential for autophagosome formation (19). To better understand the function of CD147 in autophagy, the knockdown of CD147 expression in PC-3 cells was characterized. The present data revealed that downregulation of CD147 led to increased LC3-II expression and GFP-LC3 puncta formation. These results clearly confirmed that the involvement of CD147 in the induction of autophagy and indicate that CD147 inhibits autophagy in response to nutrient deprivation.

The pathways involved in autophagy progression are complicated and include the PI3K/Akt/mTOR pathway that suppresses autophagy. In the present study, the potential involvement of the PI3K/Akt/mTOR signaling pathway in CD147-induced autophagy signaling was investigated. Western blot analysis revealed significantly decreased levels of p-Akt and p-mTOR and increased levels of LC3-II in CD147 shRNA-transfected cells, indicating that the PI3K/Akt/mTOR pathway may be involved in the regulation of autophagy by CD147. In addition, it was observed that downregulation of CD147 resulted in enhanced cell death, indicating that CD147 inhibits excessive cell death in autophagy.

In conclusion, the present results revealed that CD147 significantly inhibits starvation-induced autophagic death in PC-3 cells through the PI3K/Akt/mTOR pathway and prevents excessive cell death. These results provide novel insights into the function of CD147 during tumor progression.
